# Recent Advances on Early Detection of Heat Strain in Dairy Cows Using Animal-Based Indicators: A Review

**DOI:** 10.3390/ani11040980

**Published:** 2021-04-01

**Authors:** Hang Shu, Wensheng Wang, Leifeng Guo, Jérôme Bindelle

**Affiliations:** 1 Agricultural Information Institute, Chinese Academy of Agriculture Sciences, Beijing 100086, China; shuhang@caas.cn; 2 AgroBioChem/TERRA, Precision Livestock and Nutrition Unit, Gembloux Agro-Bio Tech, University of Liège, 5030 Gembloux, Belgium; jerome.bindelle@uliege.be

**Keywords:** heat strain, heat stress, dairy cows, early detection, measurement, threshold

## Abstract

**Simple Summary:**

In the dairy industry, heat stress and its induced heat strain result in huge economic loss every year. To better manage heat strain in dairy cows, it is more sensible to advance the detection by using more sensitive indicators so that cooling measures can be implemented in time. With the development of sensor technologies and wireless transmission technologies, body surface temperature and respiration rate can be measured automatically through wearable devices. Lots of efforts have been made recently to develop meaningful thresholds on both physiological and environmental sides. However, the existing thresholds should be used carefully considering the differences in experimental conditions and animal information. Further studies are required to evaluate and customize thresholds based on different influencing factors. A comprehensive early detection system for heat strain based on both animal- and environment-based indicators is expected.

**Abstract:**

In pursuit of precision livestock farming, the real-time measurement for heat strain-related data has been more and more valued. Efforts have been made recently to use more sensitive physiological indicators with the hope to better inform decision-making in heat abatement in dairy farms. To get an insight into the early detection of heat strain in dairy cows, the present review focuses on the recent efforts developing early detection methods of heat strain in dairy cows based on body temperatures and respiratory dynamics. For every candidate animal-based indicator, state-of-the-art measurement methods and existing thresholds were summarized. Body surface temperature and respiration rate were concluded to be the best early indicators of heat strain due to their high feasibility of measurement and sensitivity to heat stress. Future studies should customize heat strain thresholds according to different internal and external factors that have an impact on the sensitivity to heat stress. Wearable devices are most promising to achieve real-time measurement in practical dairy farms. Combined with internet of things technologies, a comprehensive strategy based on both animal- and environment-based indicators is expected to increase the precision of early detection of heat strain in dairy cows.

## 1. Introduction

Thermoregulation is of importance for homeotherms to maintain homeothermy and homeostasis by keeping the balance between the production and loss of body heat [1]. Heat production in dairy cows mainly comes from basal metabolism, rumen fermentation, nutrient absorption, growth, lactation, gestation, immunization, and exercise [2]. Heat loss is mainly through heat transfer mechanisms: Conduction, convection, radiation, and evaporation [1,2].

Cows can maintain a balance of heat production and heat dissipation in the thermoneutral zone (TNZ), in which cows perform the lowest physiological and immune costs, and the highest productivity [1]. When the thermal environment exceeds TNZ and suppresses the efficiency of non-evaporative heat loss, evaporative heat loss, or both [3,4], various mechanisms are activated in dairy cows to dissipate excess heat and maintain homeothermy [5]. In the present review, “heat stress” represents the sum of external stressors, forcing the animal to exceed its TNZ. Environmental thermal stressors consist of ambient temperature (AT), relative humidity (RH), wind speed, and radiation, where AT plays the leading role [6]. “Heat strain” is used to represent the overall response resulting from heat stress. These two terms are separated since many internal animal factors can alter dairy cows’ sensitivity to heat exposure.

To address the question of how animals are able to cope with heat stress and their induced heat strain, the use of animal-based indicators to detect heat strain directly is of particular importance [7,8,9]. Unfortunately, the animal husbandry sector has long been limited by the lack of labor- and time-saving measurement methods for animal-based indicators [10]. Many thermal indices have been developed to reduce the multi-dimensional thermal stressors to better depict the thermal environment, and have been connected to animal-based indicators with the hope to assess heat strain indirectly with easily accessible weather data. Temperature-humidity indices (THI)-a series of highly correlated indices combining temperature and humidity-have been developed by different scientists and are used most commonly [11]. Thermal indices have already been evaluated comprehensively in other reviews [12,13]. Notably, environmental indicators have difficulties dealing with individual differences in the onset of heat strain and showing whether animals are being adequately cooled [7,14].

For decades, the onset of heat strain has long been associated with productive parameters [15]. However, production loss is more likely a result of heat strain than an early indicator [7] and usually lags the changing environment by about two days [16,17]. In fact, heat strain includes both short- and long-term responses from vasodilation that happens instantly to gradual physiological adaptation [1,5]. Recently, many efforts have been made to use more sensitive physiological indicators to help detect heat strain in the early stage [18,19]. Among all kinds of physiological indicators, body temperatures and respiratory dynamics are discussed and used most commonly [7,18].

For dairy cows, the increased heat production due to the recent progression in productivity coupled with their insufficient heat dissipation capacity makes them currently more susceptible to heat strain [1,7]. Due to global warming, dairy cows are being exposed to heat wave events more often, and the resulting economic losses are projected to increase substantially [20,21]. All this information emphasizes the importance of improving the early detection of heat strain in dairy cows to avoid the subsequent reduction in production and welfare, and the increase in disorders and mortality, etc.

Precision livestock farming (PLF) aims to provide real-time monitoring for significant issues so that interventions can be implemented immediately [22]. In light of this, the demand for non-invasive, continuous, remote, accurate, real-time, and ultimately automatic measurements for physiological indicators of dairy cows has been more and more valued for heat strain detection [5,7,9,14,23]. Recently, many new methods have been developed to measure heat strain-related data [10,24]. On the basis of measurement, a series of thresholds are naturally developed to quantify and assess heat strain. 

To get an insight into the early detection of heat strain in dairy cows, the present review focuses on the recent efforts developing early detection methods of heat strain in dairy cows based on body temperatures and respiratory dynamics. The primary objective was to summarize the progress on the measurement methods, determining the most feasible method. The secondary objective was to summarize the existing thresholds from both physiological and environmental viewpoints, aiming to find the most sensitive indicators for the future development of early detection methods in the setting of practical dairy farms.

## 2. Detection of Heat Strain Based on the Most Commonly Used Animal-Based Indicators

The methods for obtaining heat strain-related data can be divided into two categories, i.e., direct measurements [9,10] and predictive modeling [11]. The predictive modeling is not the focus of this review, but still, it is mentioned when describing the relationship between candidate indicators. To assess and quantify heat strain, critical thresholds on both environment and animal sides have been developed. Therefore, for every candidate indicator in this section, the latest measurement methods and thresholds were discussed.

### 2.1. Core Body Temperature

Core body temperature (CBT) is the most commonly used animal-based indicator of heat strain in dairy cows, in which rectal temperature (RT) plays a leading role [18]. Normally, the range of CBT of homeotherms is very narrow with a circadian rhythm due to the effort of the thermoregulation [25]. When cows are heat-stressed, their CBT increases abnormally if they fail to dissipate excess body heat. The latest measurement methods and thresholds of CBT are listed in Table 1 and Table 2, respectively.

#### 2.1.1. Rectal Temperature (RT)

Traditionally, RT is measured manually with a digital thermometer inserted 9 cm into the rectum for calves and 15 cm for the cows [60]. This procedure is both time- and labor-consuming and cannot achieve a measurement in real-time [34]. More importantly, this invasive operation would cause additional stress to cattle, biasing the results. Gaughan et al. [61] used a temperature data logger (Smart Reader 8, ARC Systems, Brisbane) to continuously measure temperature rectally. Spiers et al. [27] used a stainless-steel thermistor probe attached to a recorder (Cole Parmer Instruments, Chicago, IL, USA) to measure RT. Reuter et al. [28] designed a device consisting of an indwelling data logger supported by a custom-fabricated aluminum tail harness to achieve an automated measurement for RT. However, due to the potential damage to the tail and the influence of fecal temperature, this device is not widely used [9,35]. Debnath et al. [30] achieved a real-time measurement for RT in dairy cows using a radiofrequency-based digital thermometer. Lees et al. [29] developed an intra-rectal device to measure RT continuously for 23 h in grazing heifers. This device consists of a data logger (iButton DS1922L, Maxim Integrated, San Jose, CA, USA) and a soft polyethylene piping. As summarized by Burfeind et al. [62], the measurement of RT could be biased by the type of thermometer, the depth into the rectum, and the operation itself.

A daily temperature circadian rhythm in cattle older than two months was observed with RT at dusk up to 1.4 °C higher than that in dawn [60]. It is noted by Tresoldi et al. [36] that RT measured a few times a day may result in a relatively lower daily average, especially when measuring only once in the morning. Except for circadian rhythm, many heat-producing events (e.g., feeding, exercise, estrus) would lead to an increased RT [30,60]. Therefore, it is of importance to avoid potential bias from these interfering events when determining the onset of heat strain.

Many studies treated environmental indicators (e.g., THI) as a categorical variable to test the RT differences among different levels of heat exposure [26,63]. Other studies adopted similar methods by separating the records into different levels according to climate conditions (e.g., hot versus moderate, summer versus spring) [34,64]. These methods could find a strong association between RT and environmental parameters, but could not obtain accurate environmental and RT thresholds.

In a study using 45 high-producing Holstein cows, Li et al. [47] applied a broken-line model and determined the threshold of RT as 38.6 °C when heat strain was triggered at a THI of 70. Similarly, Pinto et al. [19] demonstrated a slightly lower threshold of RT (38.4 °C) at the same THI of 70 using 139 multiparous lactating Holstein-Friesian cows. However, these two studies were inconsistent in the hypothesis of normal RT. The former assumed normal RT as a plateau (i.e., a constant), while the latter used a line with a slight slope to fit normal RT. In theory, it is more reasonable to regard normal RT as a range normally distributed around a constant due to the existence of the thermoregulatory system.

Dalcin et al. [48] also used broken-line models with plateaus representing normal conditions to explore thresholds in 38 lactating cows from various genetic groups (½, ¾, and pure-bred Holstein). The black globe-humidity index (BGHI) was used to describe the thermal environment. The BGHI thresholds 76.44 and 73.51 were determined for ½ and ¾ Holstein, respectively. Corresponding RT thresholds were around 38.2 and 37.8 °C. No threshold was derived for pure-bred Holstein due to the high linear correlation with BGHI. As the BGHI stayed higher than 72 during the entire experiment, it can be inferred that the BGHI threshold for pure-bred Holstein could be somewhere lower than 72. These results indicate that ½ Holstein cows were most heat-tolerant, while pure-bred Holstein cows were most vulnerable. The plateaus were also applied by Dado–Senn et al. [49] when determining thresholds of various physiological indicators in preweaning dairy calves. Limited attention has been paid to heat abatement in dairy calves due to their larger surface-to-mass ratio. Unexpectedly, a THI threshold (67) for RT was determined for calves under shade without fans.

#### 2.1.2. Vaginal Temperature

Vaginal temperature (VT) is another representative CBT. The traditional measurement of VT is very similar to that of RT and many efforts have been made to achieve automated measurements. Various temperature data loggers used in conjunction with support devices have been fixed into cows’ vaginas. A plastic anchor was first introduced as a support device by Hillman et al. [65] and further validated by Lee et al. [31]. It is demonstrated that long-finger plastic anchors were superior due to better stability when inserted in the vagina [31]. However, the application of these anchors may be difficult due to inaccessibility [35]. The most popular way is to use a modified vaginal controlled internal drug release insert, in which various data loggers are placed [32,33,35]. The prices of data loggers vary distinctly. When considering elevated VT thresholds, inexpensive data loggers failed to correctly classify animals compared with an expensive and high-accurate data logger [36]. Notably, all these abovementioned methods cannot monitor VT in real-time due to the lack of wireless transmission [35].

Sakatani et al. [38] achieved wireless transmission of VT data using a temperature sensor (Gyuonkei, Remote Inc., Oita, Japan) in conjunction with Wi-Fi technology. More recently, Wang et al. [39] designed a wireless measuring system consisting of an indwelling device equipped with temperature sensors (ADT7320, Analog Devices Inc., Norwood, MA, USA), a data collector, and a computer system.

All the indwelling devices for measuring VT can only be placed in the vagina for up to 24 days (around a week in most applications) [31,36] due to the increased risk of irritation and infection [35]. Thus, long-term monitoring cannot be carried out and only short-term monitoring is meaningful for research purposes. In addition, the temperatures recorded vaginally may be affected by various factors, e.g., logger displacement, lying [33]. In addition, the requirement of an insert may hurt cow welfare and pregnancy, which is still under assessment.

Due to close anatomical proximity between the vagina and the rectum [66], VT has been regarded to be as reliable as RT in measuring the severity of heat strain [33]. VT is highly correlated with RT with a correlation coefficient up to 0.97 and the measured values are very close, especially when measuring with the same temperature loggers [33,35,67,68]. This correlation is highly biased by the accuracy of the devices, as the digital thermometers used for RT measurement are supposed to have higher accuracy than most of the data loggers used for measuring VT [33,36]. Mean VT was found higher than mean RT which might be due to the fact that the data logger measuring VT was inserted into the vagina deeper than the thermometer measuring RT [34]. Due to the access to continuous measurement, VT is better at describing circadian rhythm than RT [33]. To reflect daily average CBT accurately, VT should be measured every 120 min or more [36].

The relationship between VT and the thermal environment has been demonstrated by many studies that VT was higher during the high THI period [26,34,37,51]. More importantly, Kaufman et al. [37] reported that VT showed stronger relationships with THI compared with RT, and therefore may be a better indicator determining heat strain in dairy cows. The vagina has abundant blood flow, making VT more sensitive to the changes in CBT than RT [35]. Nabenishi et al. [51] reported that the normal mean daily VT of 11 healthy multiparous nonpregnant Holstein-Friesian cows was stable around 38.5 °C and began to rise when THI reached 69. Furthermore, a threshold for VT (38.9 °C) was observed by Hillman et al. [52] according to the critical point where dairy cows began to stand and seek cooling. Using a broken-line model, a THI threshold (70) for VT was determined for eight lactating Holstein cows by Atkins et al. [53].

#### 2.1.3. Ruminal and Reticular Temperature

The rumen and the reticulum are also locations of interest to measure CBT. Many boluses have been designed and applied to measure ruminal and reticular parameters (e.g., temperature, pH) and many of them have already been commercially available [24,69,70,71,72]. A rumen or reticulum bolus is a configured pill placed in the reticulum or the junction between the rumen and reticulum, consisting of a temperature sensor, a telemetry system, and a battery [9]. Hicks et al. [69] first measured the temperature ruminally using the CorTemp sensors (HTI Technologies, Inc., Palmetto, FL, USA) in three cows. Initially, this method required a fistula or an implant surgery, which compromised animal welfare. At present, boluses are administered orally via a customized bolus applicator or a balling gun [68,73,74]. Rumen boluses have recently been customized for dairy calves to monitor infections, but whether these devices can be used for heat strain detection in dairy calves remains unknown [75]. With the development of wireless and battery technology, the new minimized bolus can work for more than 600 days [42]. 

Rumen temperature (RUT) and reticular temperature (RET) have been measured with the hope to develop a continuous and less invasive method for monitoring CBT than RT or VT [24,69,71]. The correlation coefficients between RUT and RT vary greatly among studies from 0.34 [70] to 0.92 [76]. The correlation coefficients between RET and RT range from 0.50 [77] to 0.645 [71]. 

The temperatures recorded in the rumen and reticulum are greater than RT by approximately 0.5 °C due to the heat produced by rumen microorganisms [71]. A drinking bout can also decrease RET by 9.2 °C and it would take up to 3.5 h to return to the original temperature before drinking [54,71]. Given that the reestablishing time depends on both temperature and amount of water drunk, it is possible to use it to predict water intake, which also plays a part in heat strain in cattle [78]. To mitigate the impact of water intake on RET, Ammer et al. [68] calculated median and mean RET for 2 h preceding RT and VT measurement and demonstrated that the median RET was more highly correlated to RT and VT (r = 0.48, 0.53) compared with mean RET (r = 0.43, 0.46) and single RET (r = 0.40, 0.48). Since calves drink less cool water than adult cattle and the milk or milk supplements are preheated, the intake of water may not bias RET as much as for adults [75].

Many previous studies demonstrated a correlation between RUT, RET, and ambient thermal environment [54,71,79,80]. Notably, Prendiville et al. [70] found that AT correlated more with RUT (r^2^ = 0.55) than with RT (r^2^ = 0.29), indicating RUT was more sensitive to thermal changes. Liang et al. [79] reported that daily RET remained stable around 40 °C until daily AT exceeded the upper critical temperature, and the increasing rate of daily RET increased with AT. However, no threshold was given in their study. Ammer et al. [54] divided THI into 4 classes: <60, 60 to 65, 65 to 70, and ≥70, and reported that the daily median RET of 28 Holstein-Friesian dairy cows with an average milk yield of 35.2 kg/d remained unchanged at 39.2 °C when THI was lower than 65 and increased to 39.3 °C (*p* < 0.001) when THI was between 65 and 70, and further increased to 39.4 °C (*p* < 0.001) when THI ≥70. These thresholds are not accurate since THI was treated as a categorical variable. Studies on the heat strain threshold of RUT, RET, as well as the corresponding environmental indicators, are very limited.

#### 2.1.4. Tympanic Temperature

Tympanic temperatures (TT) were measured in beef cattle through data loggers placed in the ear canal [81,82,83]. This method was also used in dairy cows to measure heat strain [84,85]. However, several limitations may affect its further application in practice. Firstly, cows behave uncomfortably to these foreign objects and potential ear infections may occur following the installation, especially over a long-term use [66]. Secondly, the probes need to be properly located near the tympanic membrane, and any dislocation could result in inaccurate readings [9,70,86]. To date, wireless temperature sensors that can be fixed in the ear canal have already been commercialized [86,87]. It has been reported that the latest model of these temperature-sensing ear tags has an accuracy of ±0.1% in °F [43]. In addition, for instant use, an infrared ear thermometer provides a reading of TT in one second [44].

Many efforts have been made to use TT as an indicator for heat strain in beef cattle [83,88]. Bergen and Kennedy [66] found that TTs were consistently lower (*p* < 0.01) but highly correlated (r = 0.77) with VTs during the test days in nine beef heifers. Prendiville et al. [70] reported that the temperatures recorded in the rectum through a digital thermometer were not statistically different from those recorded wirelessly in the ear canal (*p* > 0.05). In another beef cattle study, Richeson et al. [87] used a heat strain threshold of 39.8 °C for TT. However, this threshold was directly obtained from the preexisting threshold for RT. Very few studies focus on TT in dairy cows. Myers and Henderson [89] found that TT readings were lower than RT consistently in both Holstein calves and cows. Jara et al. [85] reported that the TT of 15 multiparous Holstein-Friesian dairy cows was highly associated with thermal indices, especially with the comprehensive climate index (CCI). However, no threshold was given in this study.

Due to the anatomical proximity to the hypothalamus [66], some researchers argued that TT is more sensitive and responds earlier by as fast as 18 min to changing AT compared with RT or VT [8]. Notably, TT is the only validated indicator of CBT in calves [44].

#### 2.1.5. Subcutaneous Temperature

Subcutaneous temperature (ST) is also a candidate to represent CBT since heat-stressed cows would drive more blood from the core to the peripheral body parts [1,41]. In general, measuring temperatures subcutaneously requires the use of implantable sensors and wireless transmission. The ST can vary among different implantation locations and many efforts have been made to determine the best locations for measuring accurate CBT in cattle [40,90,91]. Reid et al. [91] used implantable radiofrequency identification (RFID) microchips measuring temperature in three sites, subcutaneously at the base of the ear, posterior to the poll, and beneath the umbilical fold. They demonstrated that the temperatures at the base of the ear correlated most with RT with coefficients of 0.30 during heat stress and 0.20 during TNZ. Similarly, Chung et al. [41] designed a real-time measurement for ear base temperature by using implantable sensors and wearable RFID scanners in conjunction. In addition, they utilized RFID technology, long-range wireless technology, and Wi-Fi technology to develop a wireless communication network aiming to increase efficiency and lower human involvement. Furthermore, a long short-term memory network was applied to predict VT by using THI and ST. Their results show that the correlation coefficients between temperatures recorded under the ear base and in the vagina of three dairy cows ranged from 0.58 to 0.85. The selected machine learning algorithm could predict CBT better than linear regression with a lower root mean square error (0.081 °C versus 0.137 °C). 

Lee et al. [90] measured ST in seven Holstein steers using button-shaped data loggers (iButton DS1922L, Maxim Integrated, San Jose, CA, USA) in three sites (lateral neck, upper, and lower scapula). Due to the lack of wireless transmission, temperatures recorded were not available until recovered from an explant surgery. Their long-term results (during a whole summer and winter) show that STs were 1.39 to 1.65 °C lower than the RT and might have been influenced by AT and animal stillness; among three test locations, the ST recorded near the upper scapula had the smallest mean difference from RT. In a recent study of dairy calves [44], temperatures recorded subcutaneously in three sites (the ear scutulum, the upper scapula, and intramuscularly in the trapezius muscle of the neck) were found very different from RT. Iwasaki et al. [40] implanted wireless thermistor sensors based on Bluetooth^TM^ 4.0 in more locations to find the best location for measuring CBT. Specifically, three endoceliac and seven subcutaneous locations were selected, and machine learning was further applied to predict CBT from STs. The results show that ST under the tail base was a good indicator due to its high correlation with RT (r = 0.62) and the correlation coefficient could be further increased to 0.93 by introducing a back-propagation type neural network with the input of seven STs and AT.

Basically, previous studies in ST aim to predict gold standard CBT (i.e., RT and VT), and some locations very related to AT were considered to interfere with the prediction. In fact, the STs of the peripheral parts respond to the thermal changes earlier than RT or VT. To date, very few studies have attempted to use ST directly as a more sensitive indicator of heat strain. In addition, although adverse events were not reported by almost all relative studies, very few of them observed the impact of implantation on health over a long-term period. It is also unclear whether the implanted sensors can be recycled from slaughterhouses or have any impact on food safety. The cost and complexity limit their further applications to research instead of practical production.

#### 2.1.6. Other Core Temperatures

Milk temperature (MT) has been proposed as a potential indicator for heat strain in dairy cows. With the development and application of automatic milking systems, the MT per milking per cow can be collected and stored automatically.

MT was reported to be correlated with VT in 31 dairy cows during a week (r = 0.52) [92]. Igono et al. [93] reported that MT measured in the clawpiece at 1600 pm correlated most with RT measured at 1630 pm right after the milking in Holstein cows housed in shade (r = 0.875) and shade plus water spray and fan (r = 0.894). In addition, the MT was reported to be significantly influenced by the maximum AT [94] and THI [95]. All of these contribute that MT is a good indicator of heat strain in dairy cows. More importantly, the effect of THI on MT differs by production level [95]. Specifically, MT increased with the increase of THI and the effect was more obvious for high-producing cows, indicating the importance to group the cows by their productivity. Ji et al. [55] first divided the cows into different age, body mass, and day in milk subgroups according to production level, respectively, and then used segmented regression models to obtain a series of thresholds. In a study where the fever was defined as VT higher than 39.5 °C, a threshold for MT (38.7 °C) had the highest combination of sensitivity (0.77) and specificity (0.66) [92].

Although MT is a time- and labor-saving way to detect heat strain in dairy cows, it is only applicable to lactating cows [90]. At the same time, MT may be less effective to inform cooling decisions in the early stage due to less frequent measurements. Instead, MT is more like a quality controller to assure that the intensive cooling protocol implemented before milking truly works. In addition, bringing cows to the milking parlor could cause additional stress resulting in an increased body temperature [96].

Finally, the nasal submucosal temperature measured through biothermal sensors was highly correlated with THI (*p* < 0.01) and increased more rapidly than RT [97]. However, no more efforts were found, which may have been due to the huge fluctuation.

### 2.2. Body Surface Temperature

When dairy cows first feel discomfort during heat exposure, vasodilation allows more blood to divert from the core to the skin, which increases the body surface temperature (BST), allowing for increased heat exchange with the environment [41]. Although CBT seems to be the most valuable parameter for assessing heat strain and only 6% of the studies considered BST [18], the application of BST has been increasingly mentioned in recent years as its non-invasive measurement is more in line with the requirements of animal welfare and PLF [98,99,100]. The latest measurement methods and thresholds of BST are listed in Table 1 and Table 2, respectively.

There are multiple ways to measure BST, among which infrared thermometry (IRT) is the most popular one due to its low cost and the fact that it does not require direct contact [101]. Infrared thermometry can be used in both portable and fixed ways. For portable use, an infrared camera or infrared gun can be held to measure data according to users’ needs [37,45,102,103]. However, manual operation limits the frequency of data collection, and therefore may be not applicable to achieve real-time measurement in dairy farms. For fixed use, an infrared camera can be fixed at a specific location (e.g., milking parlor, calf feeder, water station) [46,104,105]. In the study by Hoffmann et al. [105], the regions of interest were manually defined. The introduction of the feature tracking technique helped automatically locate the regions of interest, and therefore increased the efficiency [46]. Using RFID technology, an automated and wireless collection of IRT data from cattle was successfully achieved [104]. Generally, high-accuracy infrared cameras are relatively expensive, whereas affordable infrared guns may be not reliable in results. In addition, the results of IRT are affected by the dirt on the surface, the skin fold, the direct sun exposure, the distance and angle to the object animal, etc. [7]. It has been reported that BSTs from IRT measured at eyes (r = 0.58) [106] and forehead (r = 0.557) [45] have the highest correlation with RT. More importantly, the presence of liquid (e.g., sweat) along the surface of the skin will significantly underestimate the real BST [107], indicating that additional image processing is needed when animals are under heat stress.

In addition to IRT, BST can also be measured through wearable devices. Wearable devices equipped with wireless transmission and positioning technologies are of particular importance in the setting of grazing pastures due to the ability to remotely monitor the thermal comfort of individual animals or the entire herd. Data loggers (iButton DS1922L, Maxim Integrated, San Jose, CA, USA) were placed on the skin above the tail vein opposing the rectum using flexible tape [108]. Thermocouples and thermistors were also fixed to the skin to measure BST [109,110,111]. The results of the thermistors (EU-UU-VL5–0, Grant Instruments, Cambridge, UK) and data loggers (iButton DS1922L, Maxim Integrated, Sunnyvale, CA, USA) were demonstrated to have a very good agreement when measuring BST [107]. However, wearable devices are difficult to fix on the skin surface and prone to displacement, and the results recorded were easily affected by the hair and air movement [40]. To minimize the influence of wind and hair, Kou et al. [110] designed an automated measurement for BST by using a shell shape to fix the thermistor appropriate to the metatarsus of the hind leg with less hair.

To further estimate the relationship between BST and other physiological indicators, some studies have intended to predict standard indicators (i.e., RT, respiration rate (RR)) based on BST [110]. Recently, machine learning models (e.g., artificial neural networks (ANNs)) have been used to enhance the predictive ability from BST to RT and RR in beef cattle and dairy cattle [112,113]. Sousa et al. [112] yielded a better coefficient of determination of 0.72 from an ANN model compared with a linear regression model (0.57) when predicting RT. Similarly, Pacheco et al. [113] also found that ANN-based models yielded higher coefficients of determination (0.71 for RT and 0.74 for RR) than the linear regression models (0.55 for RT and 0.69 for RR). These efforts provide powerful tools to predict core parameters which are important physiological indicators not only in the detection of heat strain.

To better and earlier manage heat strain, some studies have attempted to develop thresholds based on BST directly. Unlike CBT, BSTs vary apparently with the ambient environment and no plateau stage exists. Therefore, attention should be paid to the point where BST begins to rise more rapidly. Peng et al. [45] used segmented regressions developing thresholds of THI for forehead temperature. Mean and maximum forehead temperatures began to increase rapidly when THI reached 71.4 and 66.8, respectively, which were lower than the THI threshold for RT (74.1). The corresponding thresholds of mean and maximum forehead temperatures were 30.05 and 30.34 °C, respectively. Kim et al. [56] developed a series of THI thresholds for five body regions (eyes, hindquarters, nose, part of horns, and ears) based on data from eight Hanwoo heifers. The THI thresholds ranging from 65 (eyes) to 70 (hindquarters) were obtained as the point where BSTs surpassed CBT. Dado–Senn et al. [49] also utilized a segmented regression to develop the threshold of BST in preweaning dairy calves. However, no breakpoints were found due to the strong linear relationship to THI and the authors suggested that the breakpoint of BST could exist in a much lower range of THI.

### 2.3. Respiration-Based Indicators

Respiration-based indicators (i.e., RR, panting score (PS)) are considered to be relevant indicators for assessing heat strain in dairy cows just like RT. Indeed, panting, along with sweating, contributes to increased evaporative heat losses that are used to maintain homeothermy in cattle. More importantly, the respiratory system starts to help dissipate heat before CBTs measured rectally or vaginally begin to rise [8,61,114,115]. Respiration-based indicators may be the most appropriate indicators to early monitor heat strain in dairy cows due to high sensitivity to stressors and cost-effective measurement [115]. The latest measurement methods and thresholds of respiration-based indicators are listed in Table 3 and Table 4, respectively.

#### 2.3.1. Respiration Rate

The traditional method to measure RR is to count the movements of the flank manually and convert it into breaths per minute (BPM). Obviously, such a visual observation is both time- and labor-consuming [118]. In addition, the interaction between people and cows when measuring may bias the result [116]. Many efforts have been made recently with the hope to design automatic measurement methods for RR. Generally, these new methods can be categorized into wearable and non-wearable devices.

Most of the automatic measurement methods are wearable devices, which are mounted to a specific location of the cow (e.g., face, flank, chest, neck). Respiration-related electrical signals are collected based on different methodologies and further converted into RR. Eigenberg et al. [117] developed a thoracic belt for cows based on a commercially available device for humans. The device consists of a force transducer deriving electrical signals from rib cage movement, and a small battery-powered micro-computer further converting the signal into RR. The result of this device differed from the result of manual counting by 2–3 BPM, probably due to shallow breathing and other confounding movements. Its memory could collect RR data for four consecutive days under the condition of collecting 1 min every 15 min. In addition, the device was reported to slip off frequently [118]. More recently, Atkins et al. [53] designed a device for research based on a similar methodology as Eigenberg et al. [117]. Sensors were equipped on eight lactating Holstein cows to detect the breathing-related abdominal expansion. Two methods (peak-to-peak and fast Fourier transform) were used to derive RR from the signal. Milan et al. [116] developed a halter based on thermistor to measure RR according to the temperature changes around nostrils during breathing and found that the results of the device were slightly lower than that of manual counting in four out of five cows. However, they did not validate its reliability when cows were heat-stressed, nor did they achieve a real-time measurement. It can be inferred that this method may be of limited use when the AT is close to exhaled air temperature since no pulses in temperature would then be detected. Strutzke et al. [118] also designed a halter that can automatically detect the RR of cows based on the pressure difference between inhalation and exhalation of air. This method was also expected to provide information about breath depth. A wireless local area network was applied to achieve wireless transmission. The results show no significant difference (*p* > 0.05) between the manual counting and test device when cows were dozing or lying. However, the device tended to overestimate the RR with a mean difference of 1.4 BPM when the cows were standing (*p* < 0.05). The authors explained that this could be due to incomplete manual counting. Their device also had many limitations, e.g., short battery life, loss of devices, and the unknown reliability of the device in hot conditions as well. In addition, although there was no adverse event observed during the study, the need to insert a flexible tube 10 cm deep into the nasal cavity may cause some problems since cattle like to lick noses [126]. A facial mask equipped with an indirect calorimetry system is very important for research purposes due to the ability to measure comprehensive respiratory characteristics, including RR, tidal volume, and the composition of exhaled breath [122]. Recently, an accelerometer-based neck-mounted collar was further developed to measure RR [121]. Using Fourier transform, the respiration could be distinguished from rumination, and RR could be obtained directly from the frequency domain. In addition, the amplitude may be indicative of breath depth. More recently, an acoustic method was developed by using an MP3 audio recorder mounted to the cow’s halter [119]. No significant difference (*p* > 0.05) was found between RR measured by the acoustic and visual method in both heifers and lactating cows. However, the RR was retrospectively analyzed after data collection and the battery could only last up to two days of continuous work. To sum up, considering that almost no wearable device was reported to interfere with cows’ normal behavior, wearable devices are expected to continuously measure RR in real-time in dairy production if battery life, wearing stability, and remote wireless transmission can be further improved.

Among the non-wearable devices of measuring RR, the temperature changes around nostrils during breathing can also be captured by hand-held IRT cameras and this method has already been validated in both calves and adult dairy cattle [127,128]. The mean difference between IRT and manual counting was 0.83 BPM in adult cattle and 0.02 BPM in calves. More recently, Jorquera–Chavez et al. [46] used fixed thermal camera and computer vision algorithms to calculate RR based on the changes in pixel intensity within regions of interest. Again, the systemic defect that the AT and exhaled air temperature were difficult to distinguish in a hot environment makes this method only suitable for specific cool indoor areas, such as milking parlors. Pastell et al. [120] designed a novel method based on a laser distance sensor. The laser rangefinder (L-Gage LT3, Banner, Minneapolis, MN, USA) was fixed on the side of the cow and the RR was calculated from the change in the distance to the flank detected when the cow was breathing. The mean difference was 6 BPM when the detection frequency was set to 100 Hz. To sum up, non-wearable devices measuring RR are not mobile, and therefore would be better to use in the scenarios where cows are separated and fixed in specific areas (e.g., milking parlors, calf feeder). 

A non-heat-stressed cow has a RR range of 26–50 BPM [121] and heat stress would significantly increase RR [26,129]. Eigenberg et al. [117] reported that RR fluctuated less significantly during the thermoneutral period (18 ± 7 °C) (45 ± 16 BPM) compared with the heat stress period (>25 °C) (78 ± 26 BPM), indicating a nonlinear response of RR to AT. A consistent trend was found by Li et al. [47] in a field study with 45 healthy high-producing Holstein cows in Beijing where the AT averaged 22.0 °C (9.5–30.8 °C) and RH averaged 61.8% (37.9–82.2%). They found a greater R^2^ (0.916) after introducing a quadratic term of AT. A threshold of RR (48 BPM) where cows began to enter heat strain was further determined and the corresponding AT was 20.4 °C. Pinto et al. [19] also found RR increased faster with a higher positive slope as THI increased. Broken-line models were applied and RR thresholds of 37 BPM for standing cows and 39 BPM for lying cows were determined by choosing the models with the lowest Akaike information criterion. The corresponding THI thresholds were 70 and 65 for standing and lying cows, respectively. A lower RR threshold (60 BPM) was determined for lactating dairy cows based on the occurrence of characteristic panting behaviors (slight panting, apparent chest movement, no drool, and closed mouth) [123]. Using segmented regression models, Dado–Senn et al. [49] determined THI thresholds (65, 69) for RR in preweaning dairy calves under shade only and shade plus fans, respectively. In the study by Dalcin et al. [48] where similar broken-line models were used, BGHI thresholds (73.61, 72.29) for RR were determined for ½ and ¾ Holstein cows, respectively. A recent study by Toledo et al. [125] summarized 6 different dry-period experiments and concluded that RR over 61 BPM can be used as a threshold to indicate heat strain in dry cows.

An RR ceiling was reported by many studies, which could be due to a shift in the respiratory pattern from rapid and shallow breathing (reduced tidal volume) to slower and deeper breathing (increased tidal volume) [5,61,130]. This process is necessary to exhaust vaporized moisture in the lung and cope with respiratory alkalosis following prolonged panting [8]. This biphasic pattern is also supported by the panting scoring system adapted by Lees et al. [123] where RR may decrease during the most severe situation of panting. The critical CBT threshold for the shift in the respiratory pattern was estimated between 40.9 and 41.4 °C for Angus steers [130]. A close threshold (CBT of 40.6 °C) was found for Holstein cows where the probability of heavy breathing began to decrease [8]. To detect this change in respiratory pattern, it is of importance to measure both the speed (RR) and width (tidal volume) of breathing. In addition, RR must be measured every 30 min to provide an accurate reflection of heat strain [131].

#### 2.3.2. Panting Score

Panting-as an important physiological pathway of evaporative heat loss—has long been proposed to describe heat strain in dairy cows [132]. The main panting characteristics include increased RR, chest movement, drooling, mouth opened, tongue protruding, and neck extended [123]. With the accumulation of heat load in dairy cattle (manifested as a rising RR), cows first exhibited drooling, following by open mouth and protruding tongue [131]. PS has been developed and refined as a numerical scale to grade the obvious respiratory dynamics and behaviors [59,88,123,124]. A recent study utilized a score consisting of RR and various panting characteristics to assess heat strain in grazing dairy cows [133]. This score is very similar to PS, using a 0 to 4 scale, where 0 represents that the cow is under no heat strain and 4 represents that the cow is moribund. The PS per se, as a scoring system, has a good ability to assess heat strain in cattle. Existing panting scoring systems often provide corresponding RR thresholds, e.g., PS 1 (slight panting) corresponding to a RR of 60 BPM [123]. Thermal indices (heat load index (HLI) and dairy heat load index (DHLI)) have been developed based on PS due to its high association with the thermal environment [88,123].

To measure PS, trained observers would rate a cow’s respiratory dynamics according to the numerical scale, and the process would take about 10 s per observation per cow. Although measuring PS is relatively easier than RR, the inter-observer difference can be an important source of error. Accelerometer-based ear tags and neck collars have been designed to measure a series of behaviors including “heavy breathing”. The capacity of a commercially available collar has been validated to accurately reflect heat strain in dairy cows [8]. “Heavy breathing” detected by this tag is defined by characteristic movements (e.g., forward-backward heaving, increased RR, increased chest movement). Their results show that the proportion of cows breathing heavily was consistent with the change of mean CBT in both dry and lactating cows. An accelerometer-based ear tag from the same manufacturer was also validated in steers [124]. The visual measurement of PS and sensor-recorded “heavy breathing” were compared. The sensor was designed to give a single output within seven behaviors for every single minute, probably leading to a high false-negative rate by rating breathing heavily cows as other more prominent behaviors. Although data processing was conducted to mitigate this systematic underestimation, low sensitivities were still yielded for all PS data, PS1, and PS2 (59%, 54%, and 82%, respectively). The higher sensitivity for PS2 indicates that mild symptoms were hard to detect. In fact, the determination of “heavy breathing” in these devices is most likely to be based on a default threshold of the frequency of respiratory movements. Therefore, the original output of these devices is probably much closer to RR, and “heavy breathing” can be seen as the extended results of RR. As proposed by both Bar et al. [8] and Islam et al. [124], these accelerometer-based sensors have difficulties in detecting slight panting (PS1) and only give a binary result. Although the detection of panting or not is enough for the start of heat abatement protocol, more sophisticated classification would be more informative for customized interventions. Further studies can therefore focus on increasing the sensitivity and classification of the sensor.

## 3. Comparison and Future Development of Detection Methods

This section first compares candidate animal-based indicators from the perspectives of measurement feasibility and sensitivity to heat stress. Next, the issues that need careful attention in the process of threshold establishment are discussed and summarized. Last, a comprehensive assessment method to help with the precision management of heat strain is proposed.

### 3.1. Measurement Feasibility of Different Animal-Based Indicators

In pursuit of PLF and animal welfare, an ideal measurement method for heat strain-related data should be automatic, accurate, continuous, remote, non-invasive, low-cost, and real-time [14,99,100]. Non-contact measurements based on infrared thermometry are non-invasive to dairy cows but difficult to achieve real-time measurement at the herd level. They are more suitable for monitoring at specific locations such as calf islands, calf feeders, and milking parlors.

Indwelling devices measuring CBT rectally and vaginally cannot work for a long time due to the interference with normal physiological activities, making them more appropriate for short-term temperature monitoring. Although implantable devices can measure CBT subcutaneously, their cost and long-term safety should be further examined. Notably, ear canal sensors and rumen boluses are more appropriate for being less invasive. However, commercially available products should be further validated on the function of detecting heat strain in dairy cows.

Wearable devices are most promising since they can measure BST and RR non-invasively in real-time, and can further forecast CBT through machine learning algorithms. BST is more difficult to measure relative to RR due to various influencing factors. Currently, few wearable devices on the market can measure BST and RR. Some commercially available accelerometer-based ear tags and collars can measure “heavy breathing” [8,124]. In practical farms, there is a need for measuring comprehensive respiratory dynamics including both frequency and depth. These functions should preferably be integrated into existing commercial devices. In the future, the battery life, biocompatibility, stability, remote transmission, and accuracy of devices should be improved.

### 3.2. Sensitivity of Different Animal-Based Indicators to Heat Stress

As discussed above, many candidate physiological indicators may reflect heat strain in dairy cows. However, these animal-based indicators play different roles in different stages of coping with heat stress. Some of them (e.g., BST and RR) represent the effort made by the thermoregulatory mechanism while CBT is more like the result of thermoregulation [134].

From a thermodynamics point of view, homeotherms take two ways to dissipate body heat, which are sensible heat loss (non-evaporation) and latent heat loss (evaporation). The former consists of conduction, convection, and radiation. The latter consists of respiratory evaporation and cutaneous evaporation, where cutaneous evaporation accounts for up to 80% of total latent heat loss in lactating Holstein cows raised in a tropical environment [4]. However, cutaneous evaporation is more difficult to measure in practice than respiratory evaporation. When AT increases beyond the upper critical temperature of TNZ, the non-evaporative ways of heat loss appear to be far less efficient [1]. When the AT is close to or higher than 32 °C, Holstein cows even start to gain heat from the ambient environment through sensible heat transfer [3]. As the thermal environment continues to deteriorate, cows can succumb to hyperthermia with an increased CBT at some point, where they fail to maintain thermoneutrality [1].

From a physiological point of view, when the AT rises in the first place, homeotherm will take three main physiological procedures to increase heat dissipation, i.e., vasodilatation, sweating, and panting. The first one is to drive more blood from the core to the skin and peripheral to increase the BST so that promote sensible heat loss. The remaining two procedures are to promote evaporation [1,5]. Only when all these preliminary efforts fail to dissipate excess body heat would CBT increase abnormally [114,125]. When the increase in BST and RR is effective to keep thermoneutrality, RT can remain unchanged [135]. 

This temporal sequence responding to heat stress is supported by many studies that BST and RR increased at lower THI threshold relative to CBT, and BST responded even before RR due to direct contact with the ambient environment [19,45,48,49]. In addition, RR started to increase approximately an hour earlier than RT [61]. Panting responded about 15 min earlier than CBT both when increasing and decreasing [8]. This evidence supports the increase in CBT as a consequence of failed thermoregulation.

Indeed, the increased energy demanded by the preliminary physiological responses (e.g., panting, sweating) in addition to the reduction in energy intake (decreased feed intake) lead to a decrease in the energy used for production [136,137]. Cooling measures are used to reduce the extra energy used by cows to maintain thermoneutrality, thereby consistently maximizing production performance. Since an increased CBT already represents failed thermoregulation, it might be reasonable to advance the detection by using more sensitive indicators so that cooling measures can be taken as early as possible. Providing cooling supports when the cow is still able to dissipate heat through its thermoregulation system may be more efficient. It has been reported that providing convective cooling when the temperature gradient from the skin to the surrounding environment was larger could better reduce heat strain and yield higher milk production [138].

Recently, there has been a trend to use more sensitive animal-based parameters as indicators [19,49]. A study by Dalcin et al. [48] argued that RR was most suitable to indicate heat strain in dairy cattle compared with RT and heart rate due to the highest sensitivity to the change in the thermal index. Amamou et al. [129] also proposed that BST and RR might be better early predictors for heat strain due to more significant variability with THI compared with RT and production traits. Since the BST is highly linearly related to AT, it may be difficult to find a valuable inflection point. Future studies should compare BST with RR to determine the best point to implement interventions.

### 3.3. Selection of Thermal Indices Suitable for Specific Environments

Although AT showed close or even better correlations with animal-based indicators than thermal indices in some studies [11,47,121,139,140], a comprehensive index is still indispensable to represent the thermal environment to which animals are exposed for most of the time when developing thresholds. Thermal indices mentioned in this review were summarized in Table 5. Obviously, their formulas vary from simple indices like THI to complex indices like CCI. For more information on thermal indices, see [12,13].

As pointed out in other reviews, there is no recognized best index at present [12,13]. In a recent study by Yan et al. [143], nine typical cattle-related thermal indices have been compared using 273 lactating Holstein-Friesian dairy cows in a naturally ventilated commercial barn in Jiangsu, China. The results show that some indices could not even reflect the response of physiological indicators (RR, VT) to heat stress, and CCI was determined as the best thermal index. Similarly, CCI was found to have the highest correlation with CBT by Jara et al. [85]. Kaufman et al. [37] found simple thermal indices like THI only had a weak correlation with physiological indicators which could be due to the lack of wind speed and other management factors. Dado-Senn et al. [49] reported that wind speed was an important predicting factor of animal-based indicators in calves exposed to fans. In addition, THI with larger weights on humidity were more suitable for use in areas with higher humidity, and vice versa [144]. Generally, different indices are suitable for specific environments, especially in places similar to where the indices were originally established. Inappropriate use of thermal indices may obscure real physiological responses. Therefore, future studies should comprehensively compare and select the most appropriate thermal index in specific environments.

An important reason for poor extrapolation is due to the fact that the thermal indices of dairy cows are mostly modeled by empirical statistical models. The lack of consideration of the heat transfer mechanism between dairy cows and the thermal environment results in insufficient performance when the thermal indices are extrapolated to a non-modeled environment [145]. The thermal index based on the rational model may have better adaptability and extrapolation due to the comprehensive consideration of the heat balance equation. However, THI are still the most welcomed thermal indices in both experimental and practical conditions probably due to easy access and acceptable performance.

### 3.4. Development of Critical Thresholds

The most commonly used models to describe the relationship between environmental indicators and animal-based physiological indicators are piecewise regression models and polynomial models. Although polynomial models or other nonparametric models (e.g., generalized additive model) may bring better fitting results, they cannot directly provide easy-to-interpret slope estimates and thresholds. In contrast, the result of the piecewise function is more explanatory and can provide more useful information, including the threshold, the slope before the threshold, and the slope after the threshold. By introducing more phases into the piecewise model, more thresholds corresponding to different severity of heat strain could be identified, providing more options for cooling.

In the previous studies, the consideration of the plateau period is quite different when using piecewise functions. The assumption of the plateau could have a great impact on the results. Li et al. [47] and Peng et al. [45] collected data from the same dairy farm and both used segmented regression to obtain heat strain thresholds for RT in dairy cows. The former assumed the existence of a plateau while the latter did not. The THI thresholds were 70 and 74.1, respectively. As the experimental condition and animal status were almost identical, it can be inferred that the introduction of the plateau period could lower the THI threshold to a certain extent. In fact, the decision should base on the changing law of specific physiological indicators. For example, it is suitable to use a plateau to fit CBT as the normal CBT of homeotherms fluctuates within a narrow range. Other physiological indicators that are more sensitive to the thermal environment, such as BST and RR, may not have a significant plateau.

In addition, the thermal condition (THI range) during the testing days is of key importance to the results. For example, Dado-Senn et al. [49] and Kovács et al. [50] both used piecewise regression models to determine the THI thresholds for RT, RR, and BST in dairy calves. However, the THI thresholds of the former were between 65 and 69, while the THI thresholds of the latter were between 82.4 and 88.1. It is mostly because that the former was conducted under a broader range of thermal exposures (THI 60–85), while the environment during the latter study was much hotter (the average THI was greater than 80 for three out of four days). Therefore, the thresholds obtained by the latter may be the points at which heat strain was aggravated rather than triggered.

Many studies only measured heat strain-related data once or twice a day, which may affect the accuracy of the threshold. If the physiological indicators change more frequently than the frequency of measurements, it is difficult to observe the real effect [28]. Continuous measurement is not only beneficial for threshold acquisition but also beneficial for early detection of heat strain. A broader range of thermal exposure and more frequent measurements are therefore needed for future studies on early detection of heat strain.

Various factors have an impact on the sensitivity to heat stress, including internal animal factors (e.g., age, breed, productivity, lactation stage, parity, body posture, circadian rhythm, previous thermal exposure, acclimation) [1,19,27] and external management factors (e.g., milking, cooling, nutrition, shading) [32,49,146]. Therefore, it is difficult to assess the heat strain status of a herd based on a generic threshold. To deal with these dilemmas, efforts have been made to adjust or customize the thresholds according to various animal and management factors. The HLI was developed for feedlot beef cattle by Gaughan et al. [88]. Genotype, coat color, health status, acclimatization, shade, days on feed, manure management, and drinking water temperature were considered when determining the HLI threshold for reference animals and further threshold adjustments. Other studies also tried to customize heat strain threshold according to productivity [55], posture [19], or cooling strategy [49]. Notably, the difference in sensitivity to heat stress manifests both in environmental and physiological levels. For instance, high-producing cows may enter heat strain in a lower THI and a higher RT [55,95]. Further studies are required to evaluate and customize heat strain thresholds based on different internal and external influencing factors that have an impact on the sensitivity to heat stress.

Most of the present critical thresholds assume that physiological indicators change simultaneously with the thermal environment and do not consider the cumulative effect of heat exposure. It is generally assumed that this accumulative effect only makes sense after the trigger point of heat strain. For the early detection of heat strain, due to the sensitivity of the selected indicators, the effect of time may be negligible. However, for the prediction of long-term effects such as milk production, the duration of heat exposure is an indispensable factor [17]. In addition, when dairy cows have long been exposed to heat stress, some of them may adapt to heat stress and can better dissipate excess body heat, manifesting as increased RR and BST, and restored milk yield [129]. Therefore, existing RR and BST thresholds may be exaggerated to some extent if lots of thermotolerant data were used for thresholds development.

### 3.5. Individual Monitoring to Help Precision Management

After obtaining the thresholds on both physiological and environmental dimensions, it is important to assess how to use them in practice. Currently, the most popular method is to evaluate the heat strain level of the entire herd indirectly through the thermal index calculated from easily accessible meteorological data, such as data from the nearby weather station, or the average value from temperature and humidity sensors placed in the barn. However, given the large differences in the microclimate to which individual dairy cows are exposed [147], a more accurate way is to measure and evaluate the microclimate by placing more sensors and dividing the barn into smaller measuring and controlling units. Through the internet of things (IoT) technology, the cooling facilities in a certain unit can be monitored continuously and the heat strain of the herd can be managed more effectively and efficiently.

Environmental indicators neither truly reflect how animals respond to the changes in the thermal environment nor show whether animals are being adequately cooled [14]. It has been reported that both CBT and RR remained high even when THI was low at night [53]. In addition, there are large differences in the sensitivity to the thermal environment among individual animals [1,27], even within the same subgroup [124]. A large amount of energy used for cooling measures may be wasted on low-risk animals. Therefore, direct monitoring of animal-based physiological indicators at the individual level should help better manage heat strain. At present, many large-scale commercial dairy farms have used smart ear tags or collars to monitor multiple physiological behaviors of individual cows. Combined with positioning technology (e.g., RFID technology), the nearby cooling facilities can be turned on automatically when a large proportion of cows breathing heavily in a small area. Such an IoT system is very promising not only because it could detect heat strain more accurately and earlier based on animal’s feeling, but also because it could save energy. In addition, the heat strain screening system in the passage to the parlor could achieve an automatic separation of heat-stressed cows [148].

Combining environmental indicators and various physiological indicators to evaluate heat strain from multiple perspectives also has a good prospect. Due to different physiological dynamics, it is possible to distinguish heat strain from other events that may lead to increased body temperature and accelerated breathing, including exercise [30], diseases (e.g., infectious disease [91]), physiological processes (e.g., estrous [149]), and management factors (e.g., bringing cows to the milking parlor [96]).

## 4. Conclusions

In consideration of the measurement feasibility and the sensitivity to heat stress, we concluded that BST and RR are the most appropriate indicators for the early detection of heat strain in dairy cows. Although the thresholds of both physiological and environmental indicators can be obtained through regression models, many studies did not give corresponding thresholds for physiological indicators probably due to the lack of real-time measurement for physiological data in actual production. The existing thresholds should be used carefully due to the differences in experimental conditions and animal information. Future studies should customize heat strain thresholds according to different internal and external factors that have an impact on the sensitivity to heat stress. With the development of wireless transmission technology, sensor technology, and battery technology, wearable devices are most promising to achieve real-time measurements in practical dairy farms. Combined with IoT technologies, a comprehensive strategy based on both animal- and environment-based indicators is expected to increase the precision of early detection of heat strain.

## Figures and Tables

**Table 1:** Summary of the state-of-the-art measurement methods for body temperatures.

Indicator	Technology	Automatic	Continuous	Real-Time	Accuracy	Reference
Rectal temperature	Digital thermometer	No	No	No	±0.1 °C	Garner et al. [26]
	A thermistor probe attached to a recorder	Yes	Yes	No	Unknown	Spiers et al. [27]
	An indwelling data logger supported by a customized tail harness or piping	Yes	Yes	No	±0.2 to ± 0.5 °C	Reuter et al. [28]; Lees et al. [29]
	Radiofrequency-based digital thermometer	Yes	Yes	Yes	±0.5 °C	Debnath et al. [30]
Vaginal temperature	Temperature probe with a long-finger anchor	Yes	Yes	No	±0.2 °C	Lee et al. [31]
	Temperature data loggers and modified vaginal controlled internal drug release	Yes	Yes	No	±0.1 to ±1 °C	Kendall et al. [32]; Vickers et al. [33]; Burfeind et al. [34]; Burdick et al. [35]; Tresoldi et al. [36]; Kaufman et al. [37]; Garner et al. [26]
	A wireless vaginal temperature device	Yes	Yes	Yes	Unknown	Sakatani et al. [38]
	An indwelling device equipped with temperature sensors, a data collector, and a computer system	Yes	Yes	Yes	Mean difference of 0.02 °C with a 95% confidence interval: −0.23 to 0.26 °C	Wang et al. [39]
Subcutaneous temperature	Implantable wireless thermometers based on Bluetooth	Yes	Yes	Yes	±0.05 °C	Iwasaki et al. [40]
	Implantable radiofrequency identification biosensors and wearable scanners	Yes	Yes	Yes	Unknown	Chung et al. [41]
Ruminal temperature	Radiotelemetric ruminal and reticular bolus	Yes	Yes	Yes	Unknown	Nogami et al. [42]
Tympanic temperature	Continuous monitoring temperature probes	Yes	Yes	Yes	±0.1%	Mahendran et al. [43]
	Infrared ear thermometer	Yes	No	No	±0.2 °C	Woodrum Setser et al. [44]
Body surface temperature	Handheld infrared camera	No	No	No	±1%	Peng et al. [45]
	Handheld infrared gun	No	No	No	±1.5 °C	Kaufman et al. [37]
	Fixed infrared camera	Yes	Yes	Yes	±2%	Jorquera-Chavez et al. [46]

**Table 2:** Summary of the thresholds of body temperatures.

Indicator	Threshold for Animal-Based Indicators	Threshold for Environment-Based Indicators	Cow Information	Thermal Condition	Reference
Rectal temperature	38.4 °C	THI ^a^ 70	High-producing Holstein-Friesian dairy cows	A continental climate with a THI range of 17.8 to 85.1	Pinto et al. [19]
	38.6 °C	THI ^a^ 70	High-producing Holstein dairy cows	Hot climate with an AT range of 9.5 to 30.8 °C	Li et al. [47]
	38.55 °C	THI ^a^ 74.1	High-producing Holstein dairy cows	Warm temperate semi-humid continental monsoon climate with a THI range of 58 to 84	Peng et al. [45]
	Unknown	BGHI 76.44	½ Holstein dairy cows	BGHI stayed higher than 72	Dalcin et al. [48]
	Unknown	BGHI 73.51	¾ Holstein dairy cows	BGHI stayed higher than 72	Dalcin et al. [48]
	Unknown	THI ^a^ 67	Preweaning dairy calves	Averaged THI of 78 with shade only in a subtropical climate	Dado–Senn et al. [49]
	Unknown	THI ^b^ 88.1	Preweaning Holstein bull calves	Hot climate with a THI range from 70.3 to 94	Kovács et al. [50]
Vaginal temperature	Unknown	THI ^c^ 69	Multiparous nonpregnant Holstein-Friesian dairy cows	THI ranged from 55.8 to 79.9	Nabenishi et al. [51]
	38.9 °C	Unknown	Dairy cows	Hot–humid climate with an average THI of 82.4	Hillman et al. [52]
	Unknown	THI ^a^ 70	Lactating Holstein cows	Unknown	Atkins et al. [53]
Ruminal temperature	39.3 °C	THI ^a^ 65	Lactating Holstein-Friesian dairy cows	Temperate climate with THI values varied from 9.6 to 84.9	Ammer et al. [54]
Milk temperature	Unknown	Dynamic thresholds using a decision tree model	Lactating Holstein cows	A subtropical climate with an ambient temperature range of 2.0 to 38.0 °C	Ji et al. [55]
Body surface temperatures: Eyes, hindquarters, nose, part of horns, and ears	Unknown	THI ^a^ 65 (eyes) to 70 (hindquarters)	Hanwoo heifers	THI ranged from 75.1 to 84.7	Kim et al. [56]
Body surface temperatures: Forehead (mean, maximum)	30.05, 30.34 °C	THI ^a^ 71.4, 66.8	High-producing Holstein dairy cows	Warm temperate semi-humid continental monsoon climate with a THI range of 58 to 84	Peng et al. [45]
Body surface temperature: Ear	Unknown	THI ^b^ 83.0	Preweaning Holstein bull calves	Hot climate with a THI range from 70.3 to 94	Kovács et al. [50]

THI = temperature–humidity index; BGHI = black globe-humidity index. ^a^ THI equation from National Research Council [57]; ^b^ THI equation from Bianca et al. [58]; ^c^ THI equation from Mader et al. [59].

**Table 3:** Summary of the state-of-the-art measurement methods for respiration-related data.

Indicator	Methodology	Technology	Automatic	Continuous	Real-Time	Accuracy	Reference
Respiration rate	Temperature changes around nostrils during breathing	Infrared thermography	Yes	Yes	Yes	8.4 ± 3.4 (mean ± SD) BPM lower	Jorquera–Chavez et al. [46]
		Thermistor sensor	Yes	Yes	No	±2 BPM for 80% of the times	Milan et al. [116]
	Chest and abdominal expansion associated with breathing	Pressure sensor	Yes	Yes	No	±2–3 BPM	Eigenberg et al. [117]
						±0.47 BPM	Atkins et al. [53]
	Pressure changes around nostrils during breathing	Pressure sensor	Yes	Yes	No	Mean difference (BPM): −0.2 when dozing; 0.2 when lying; 1.4 when standing	Strutzke et al. [118]
	Breathing sounds	Acoustic processing	Yes	Yes	No	Mean bias: 2.75 BPM	de Carvalho et al. [119]
	Distance change of abdominal movement during breathing	Laser distance sensor	Yes	Yes	No	Mean difference of 6 BPM when measurement rate is 100 Hz	Pastell et al. [120]
	Body movements due to respiration	Accelerometer-based neck collar	Yes	Yes	No	Unknown	Davison et al. [121]
	The change in respiration volume	Spirometer	Yes	Yes	Yes	Unknown	de Melo Costa et al. [122]
Panting	Panting scoring system	Visual scoring	No	No	No	Not applicable	Mader et al. [59]; Gaughan et al. [88]; Lees et al. [123]
	Body movements due to panting	Accelerometer-based ear tag and neck collar	Yes	Yes	Yes	Positive predictive value = 79%	Bar et al. [8]; Islam et al. [124]

SD = standard deviation; BPM = breaths per minute; Hz = Hertz.

**Table 4:** Summary of the thresholds of respiration-related data.

Indicator	Threshold for Animal-Based Indicators	Threshold for Environment-Based Indicators	Cow Information	Thermal Condition	Reference
Respiration rate	48 BPM	THI ^a^ 70	High-producing Holstein dairy cows	Hot climate with a T_a_ range of 9.5 to 30.8 °C	Li et al. [47]
	37, 39 BPM	THI ^a^ 70, 65	Standing and lying high-producing Holstein-Friesian dairy cows	Continental climate with a THI range of 17.8 to 85.1	Pinto et al. [19]
	60 BPM	Unknown	Lactating Holstein-Friesian dairy cows	Three summers and two winters in Queensland, Australia	Lees et al. [123]
	30 BPM	BGHI 73.61	½ Holstein dairy cows	BGHI stayed higher than 72	Dalcin et al. [48]
	45 BPM	BGHI 72.29	¾ Holstein dairy cows	BGHI stayed higher than 72	Dalcin et al. [48]
	Unknown	THI ^a^ 70	Lactating Holstein cows	Unknown	Atkins et al. [53]
	61 BPM	Unknown	Dry cows	Unknown	Toledo et al. [125]
	Unknown	THI ^a^ 65	Preweaning dairy calves	Averaged THI of 78 with shade only in a subtropical climate	Dado–Senn et al. [49]
	Unknown	THI ^a^ 69	Preweaning dairy calves	Averaged THI of 78.25 with shade plus fans in a subtropical climate	Dado–Senn et al. [49]
	Unknown	THI ^b^ 82.4	Preweaning Holstein bull calves	Hot climate with a THI range from 70.3 to 94	Kovács et al. [50]

BPM = breaths per minute; THI = temperature–humidity index; T_a_ = ambient temperature (°C); BGHI = black globe-humidity index. ^a^ THI equation from National Research Council [57]; ^b^ THI equation from Bianca et al. [58].

**Table 5:** Summary of the thermal indices mentioned in this review.

Thermal Index	Formula	Resource
Temperature–humidity index (THI)	THI = (1.8 × T_a_ + 32) − (0.55 − 0.0055 × RH) × (1.8 × T_a_ − 26.8)	National Research Council [57]
	THI = (0.35 × T_a_ + 0.65 × T_w_) × 1.8 + 32	Bianca et al. [58]
	THI = 0.8 × T_a_ + (RH/100) × (T_a_ − 14.4) + 46.4	Mader et al. [59]
Black globe-humidity index (BGHI)	BGHI = T_bg_ + 0.36 × T_dp_ + 41.5	Buffington et al. [141]
Comprehensive climate index (CCI)	$\mathrm{CCI}=T_{a}+\mathrm{Equation}\;\left(1\right)+\mathrm{Equation}\;\left(2\right)+\mathrm{Equation}\;\left(3\right)$ $\mathrm{Equation}\;\left(1\right)=e^{\left(0.00182\times \mathrm{RH}+1.8\times 10^{-5}\times T_{a}\times \mathrm{RH}\right)}\times \left(0.000054\times T_{a}{}^{2}+0.00192\times T_{a}-0.0246\right)\times \left(\mathrm{RH}-30\right)$ $\mathrm{Equation}\;\left(2\right)=\frac{-6.65}{e^{\left(\frac{1}{{\left(2.26\times \mathrm{WS}+0.23\right)}^{0.45\times \left(2.9+1.14\times 10^{-6}\times {\mathrm{WS}}^{2.5}-\log_{0.3}{\left(2.26\times \mathrm{WS}+0.33\right)}^{-2}\right)}}\right)}}-0.00566\times {\mathrm{WS}}^{2}+3.33$ $\mathrm{Equation}\;\left(3\right)=0.0076\times \mathrm{RAD}-0.00002\times \mathrm{RAD}\times T_{a}+0.00005\times T_{a}{}^{2}\times \sqrt{\mathrm{RAD}}+0.1\times T_{a}-2$	Mader et al. [142]
Heat load index (HLI)	HLI (T_bg_ < 25) = 10.66 + 0.28 × RH + 1.9 × T_bg_ − WS $\mathrm{HLI}\left(T_{\mathrm{bg}}>25\right)=8.62+0.38\times \mathrm{RH}+1.55\times T_{\mathrm{bg}}-0.5\times \mathrm{WS}+e^{2.4-\mathrm{WS}}$	Gaughan et al. [88]
Dairy heat load index (DHLI)	$\mathrm{DHLI}=1.681813\times {\left(1+e^{-\left(-8.50749+0.206159\times T_{\mathrm{bg}}+4.088399\times \mathrm{RH}\right)}\right)}^{-1}$	Lees et al. [123]

T_a_ = ambient temperature (°C); RH = relative humidity; T_w_ = wet bulb temperature (°C); T_bg_ = black globe temperature (°C); T_dp_ = dewpoint temperature (°C); WS = wind speed (m/s); RAD = radiation (W/m^2^).
